# RNAi Power Targets in Insect Pests: Beyond Functional Validation to Biopesticide Development Potential

**DOI:** 10.3390/plants15121803

**Published:** 2026-06-11

**Authors:** Momana Jamil, Shakil Ahmad, Valeria Palma-Onetto, Yanping Luo

**Affiliations:** 1 School of Tropical Agriculture and Forestry, Hainan University, Haikou 570228, China; jamilmomana@gmail.com; 2 School of Ecology, Hainan University, Haikou 570228, China; 3 Guangdong Key Laboratory of Animal Conservation and Resource Utilization, Institute of Zoology, Guangdong Academy of Science, Guangzhou 510260, China; mshakil.aup@gmail.com; 4 Departamento de Química y Medio Ambiente, Universidad Técnica Federico Santa María, Avenida España 1680, Valparaíso 2390123, Chile; valeria.palmao@usm.cl

**Keywords:** crop yields, RNA interference (RNAi), sustainable agriculture, food security, biopesticides, insect pest control

## Abstract

Global agricultural production faces unprecedented challenges due to climate crisis, biodiversity loss, and increasing population pressure, while there is a growing demand for sustainable and eco-conscious food production systems. Traditional methods of crop protection like the use of synthetic chemical pesticides are becoming less effective due to the high resistance development in major insect pests. Moreover, their overuse has raised numerous environmental concerns. In this context, RNA interference (RNAi) has emerged as a promising and environmentally friendly alternative to traditional pesticides, with a more sustainable way of managing pests. This review systematically identifies promising RNAi target gene families for insect pest control, particularly key developmental genes. The selected genes were chosen based on demonstrated RNAi efficacy in at least three different insect species, emphasizing their broad applicability and potential impact. It also discusses the translation of RNAi technologies from laboratory research to field applications. It underscores the importance of moving beyond functional gene characterization to improving the efficiency and scalability of RNAi in real-world agricultural systems. This review systematically lists RNAi target genes and delivery methods in insect pests, identifies research gaps, and supports the development of RNAi-based biopesticides.

## 1. Introduction

RNA interference (RNAi) is a highly conserved cellular mechanism triggered by the presence of double-stranded RNA (dsRNA) in the cell. After processing of dsRNAs by Dicer nucleases, the resultant small interfering RNAs (siRNAs) are bound by Argonauteproteins, which stimulates the degradation of messenger RNAs (mRNAs) by the sequence complementarity between the siRNAs and the target mRNAs [1,2,3,4]. The ability of dsRNAs to trigger the RNAi mechanism was initially described in the nematode *Caenorhabditis elegans* by Andrew Fire, Craig Mello, and collaborators who were awarded the Nobel Prize in Physiology or Medicine in 2006 for their contributions to the discovery of RNAi [5]. Since then, RNAi has been found to be widespread across most eukaryotic organisms, including protozoans, invertebrates, vertebrates, fungi, algae, insects and plants, where it plays a central role in gene regulation and antiviral defense [6,7,8,9]. Since its discovery, RNAi has become a useful reverse genetics approach that has been used to study gene function at both cellular and organismal levels [10,11].

In insects, RNAi holds great promise for pest control through the silencing of genes essential for development, fecundity, or survival [12,13]. A significant breakthrough in RNAi-based pest control was the development of transgenic maize expressing dsRNA targeting the *V-ATPase A* gene of the western corn rootworm (*Diabrotica virgifera virgifera*). Disruption of this gene interferes with vital cellular functions in the insect, leading to lethality and protecting maize plants from feeding damage [14]. Following this achievement, public and private research have advanced target gene discovery and dsRNA delivery methods for RNAi-based pest management [15,16].

RNAi-based biopesticides have emerged as an eco-friendly alternative to conventional insecticides, offering a species-specific mode of action that minimizes the risk of non-target effects on beneficial organisms [17,18,19,20]. The natural biodegradability of dsRNAs helps reduce environmental contamination by decreasing residue accumulation in soil and groundwater and limiting long-term ecological persistence [21,22].

To realize the full potential of RNAi-based pest control in the field, it is essential to identify effective target genes and optimize delivery methods across a wide range of insect pests [23]. This review addresses these challenges by systematically documenting RNAi target genes that have demonstrated efficacy in multiple insect pest species, highlighting their potential for the development of RNAi-based biopesticides. Furthermore, we emphasize the importance of improving RNAi efficiency and delivery strategies to support the transition of RNAi-based methods from laboratory research to practical field applications. Although prior reviews, such as those by Christiaens et al. (2022) and Silver et al. (2021) [15,22], have concentrated largely on the mechanistic aspects of RNAi or on biosafety considerations, a comparative and gene-focused synthesis of RNAi targets across pest species, coupled with an integration of delivery strategies and commercialization prospects, remains needed. This review provides a gene-focused synthesis that links RNAi target discovery with delivery strategies and field application. By synthesizing the current knowledge and highlighting key research directions, this review provides a foundation for advancing RNAi research as a core technology in sustainable agriculture.

## 2. Mechanism of RNAi in Insects

**Figure 1:**
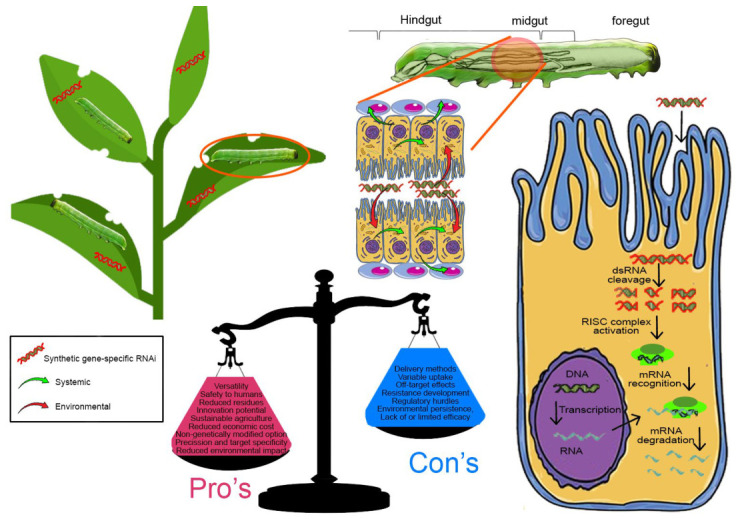
RNAi has been studied for over two decades for crop protection against insect pests. The mode of action of RNAi technology on target herbivorous insects is represented here. Cellular-level RNAi mechanisms within the insect are represented by a close-up of the cells, and double-stranded RNA (dsRNA) translocations across cells are represented by green arrows (systemic transfer) or red arrows (environmental origin) depending on their origin. The scale underscores the pros (advantages) and cons (challenges) of RNAi technology in sustainable pest management.

RNAi is a conserved biological mechanism by which small non-coding RNAs suppress gene expression by causing the cleavage of the target mRNA in a sequence-specific manner [24,25]. In insects, RNAi operates mainly through three pathways, including the siRNA pathway, the miRNA pathway, and the Piwi-interacting RNA (piRNA) pathway [26]. Although these pathways share the use of small RNAs and Argonaute proteins, they differ in their RNA precursors, Dicer requirements, Argonaute partners, biological functions, and cellular contexts. Upon cellular entry and recognition, dsRNA undergoes cleavage by the RNase III enzyme Dicer, generating 20–25 nucleotide siRNA duplexes with a two-nucleotide overhang at the 3′ end [27]. In the exogenous siRNA pathway, which is most relevant for experimentally induced RNAi and pest control applications, the long dsRNA is processed primarily by Dicer-2 into siRNA duplexes [28]. This processing is assisted by dsRNA-binding proteins such as R2D2, which facilitates siRNA loading into Argonaute-2 (Ago2)-containing RNA-induced silencing complexes (RISCs) [29,30]. Within RISCs, the passenger strand is discarded, and the guide strand directs Ago2 to bind and cleave complementary target mRNAs, resulting in rapid and sequence-specific degradation of the mRNA and subsequent silencing of the target gene expression [26]. This process enables precise functional genomics studies and targeted pest management by disrupting the expression of essential insect genes [7] (Figure 1). By contrast, the miRNA pathway is primarily involved in endogenous gene regulation. Primary miRNA transcripts are processed by Drosha and Pasha into precursor miRNAs, which are then cleaved by Dicer-1 and loaded mainly into Ago1-containing RISCs [31]. In this pathway, target recognition often involves partial complementarity and usually results in translational repression or mRNA destabilization rather than direct Ago2-mediated cleavage. The piRNA pathway differs further because it is Dicer-independent and is mainly associated with transposable element control, especially in the germline. In this pathway, Piwi family proteins, including Aubergine, Piwi, and Ago3, participate in amplification cycles such as the ping-pong cycle, thereby reinforcing genome defense [32].

Phylogenetic analyses have shown that the key genes that encode the core machinery of the three RNAi pathways have undergone diverse duplications and deletions in insects [26,33,34]. This variation is mechanistically relevant because the abundance, absence, or diversification of genes such as *Dcr2*, *Ago2*, *R2D2*, *Dcr1*, *Ago1*, *Ago3*, and Piwi-like proteins can influence dsRNA processing, siRNA loading, RISC assembly, target recognition, and ultimately the strength and consistency of RNAi responses across insect lineages [35]. Within the siRNA pathway, gene duplications have been documented for *Ago2*, *Dcr2*, and/or *R2D2* [36,37,38]. In the miRNA pathway, *Dcr1* and *Ago1* genes and four copies of *Pasha*, a cofactor of *Drosha* involved in miRNA biosynthesis, have been reported in *Acyrthosiphon pisum* (Hemiptera; Aphididae) [39]. Further, *Dcr1* and *Ago1* have been documented in *Bemisia tabaci* [40], *Ago1* in *Bactrocera dorsalis* and in *Zeugodacus cucurbitae* [32,41], *Drosha*, *Dcr1* and *Ago1* have been documented in the *Schistocerca gregaria* (Orthoptera: Acrididae) [42]. In the piRNA pathway, one *Ago3* and four *Piwi-like* genes have been reported in the *B. tabaci* genome [43]. Additionally, *Ago3* has been documented in *Drosophila melanogaster* [44] and *Tribolium castaneum* [45]. Such gene duplications, losses, and lineage-specific expansions may partly explain differences in RNAi efficiency among insect taxa [46].

## 3. Key Target Genes and Their Quantitative Summary in Different Insect Species

In this section, we present a comprehensive overview of the target genes investigated in RNAi studies across different insect species. Detailed information about all the genes, including the target insect species, knockdown effects, and dsRNA delivery methods, is provided in Supplementary Table S1.

### 3.1. RNAi-Based Key Targeted Genes

#### 3.1.1. Housekeeping and Structural Genes

**Figure 2:**
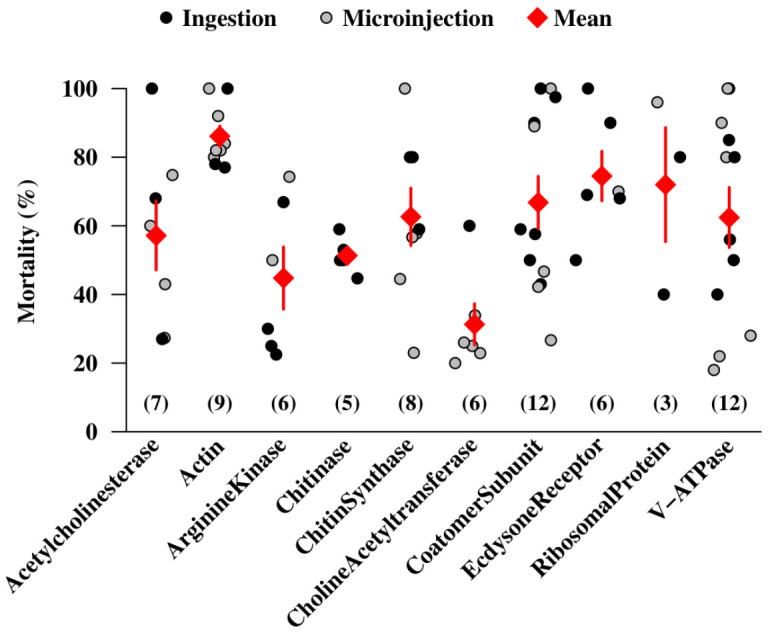
Mean mortality rate (in %) measured in insect cohorts after being treated by RNAi, either through ingestion (artificial/plant diet treated with dsRNA, transgenic plant diet, etc.; black dots) or through direct microinjection (gray dots). Each dot represents the mean value measured in an independent test (values issued from Table 1). The numbers of independent tests for each gene family are shown in parentheses. Red dots show the mean (±SE) of mortality rate values for each gene family.

In a series of studies targeting *Actin* (*ACT*) as the gene of interest for RNAi in various insect species, diverse delivery methods have been employed to achieve gene silencing and subsequent mortality of targeted insect pests. Treatment with *ACT* dsRNA exhibited 100% mortality in *Leptinotarsa decemlineata* and *Nezara viridula*, and more than 80% mortality in *Euscelidius variegatus*, *Bactericerca cockerelli* and *Cimex lectularius*, highlighting its potential as a core RNAi target for pest control (Figure 2) [47,48,49,50,51].

Targeting ribosomal proteins (RPS6, RPL26, RPL9) in *Aedes aegypti* led to a more than 88% reduction in fecundity [52], reduced ovarian provisioning and 94–99% clutch reductions in *Musca domestica* [53]. Silencing these genes resulted in adverse developmental defects and 30–100% lethality in various species, including *Leguminivora glycinivorella*, *B. tabaci*, and *D. suzukii* [54,55,56,57] (Figure 2).

*Chitin Synthase* (*CHS*) genes encode enzymes responsible for chitin biosynthesis, a key structural component of insect exoskeletons. Silencing *CHS* genes led to mortality across multiple species ranging from 50 to 70% in *Phthorimaea operculella* [58], 80% in *H. vigintioctopunctata* [59], 45% in *A. pisum* [60], 60 to 70% in *Aphis gossypii* [61], and 100% in *Anthonomus grandis* [62], *Chilo suppressalis* displayed 60% growth abnormalities [63], and 80% larval mortality in *Anopheles gambiae* [64].

RNAi targeting chitin-related genes caused significant mortality in various species, including 53% in *Chilo partellus* [65], 45% in *A. pisum* [60], 59% in *A. gossypii* [61], and over 50% in *Panonychus citri* [66], *L. decemlineata* [67] and *H. vigintioctopunctata* [68], demonstrating their potential as effective RNAi targets.

#### 3.1.2. Hormone Regulatory Pathways and Developmental Network Genes

*Ecdysone Receptor* (*EcR*) genes that encode proteins that mediate development and molting in insects are effective RNAi targets. Silencing *NlEcR-A* and *NlEcR-B* caused 90% mortality in *Nilaparvata lugens* [69], 100% in *B. tabaci* [70], 50–60% in *Helicoverpa armigera* [71], 70–80% in *Sitobion avenae* [72], and 68–70% in *Z. cucurbitae* and *Apolygus lucorum* [73,74], along with molting defects in *Blattella germanica* at adult stage [75], highlighting the *EcR* gene as a valuable candidate for pest control strategies.

RNAi targeting *Met* in various species, including *Sogatella furcifera* [76], *Harmonia axyridis* [77], *Liposcelis entomophila* [78], *Coccinella septempunctata* [79], *S. gregaria* [80], *Diploptera punctata* [81], *B. germanica* [82], and *H. armigera*, disrupted reproduction and development and increased mortality, highlighting its potential as a pest control target [83].

In *S. furcifera*, knockdown of *SfKr-h1* significantly reduced *SfVg* transcription and arrested ovarian development [84]. Similarly, in *H. axyridis,* knockdown of *HmMet* and *HmKr-h1* reduced the transcription of yolk protein genes *HmVg1* and *HmVg2*, which led to a decrease in fecundity [77]. In *H. armigera*, *HaKr-h1* silencing induced precocious larvae to pupae metamorphosis [85].

RNAi targeting COPI subunits led to increased mortality across insects, including 43% in *N. viridula* [86], 88–100% in *Brassicogethes aeneus* with injection and 43–89% with dietary exposure [87]. In *T. absoluta*, oral exposure to *dsTa-αCOP* caused 50% mortality [88], *A. aegypti* experienced 89% [89], while *D. virgifera virgifera* experienced mortality and sublethal effects in the offspring of treated females [90]. In *Colaphellus bowringi,* knockdown of *Sar1*, *Sec23*, and *Sec24* increased mortality by 27–42% [91], while *H. vigintioctopunctata* and *Z. cucurbitae* exhibited larval and adult mortality following oral dsRNA exposure [92,93], confirming COPI as a promising target for pest control (Figure 2, Table S1).

#### 3.1.3. Energy Metabolism- and Neurotransmission-Related Genes

In the exploration of *Vacuolar ATPase* (*V-ATPase*) genes as a target for RNAi across diverse insect species, the studies showcase a range of delivery methods and outcomes. Silencing *V-ATPase* caused 50–100% mortality in *Henosepilachna vigintioctopunctata*, *Locusta migratoria* and *Liriomyza trifolii* [94,95,96]. Mortality rates ranging from 18 to 80% were also reported in several species, including *Pectinophora gossypiella*, *Sphenophorus levis*, *Cimex lectularius*, *Periplaneta fuliginosa*, *B. tabaci*, and *Drosophila suzukii* [56,57,97,98,99].

Studies have shown that targeting the *Arginine kinase* (*AK*) genes significantly increased adult mortality, and greatly reduced fecundity and fertility by oral feeding of dsRNA in *Phyllotreta striolata* [100], 74% mortality and 60% decreased hatchability in *Culex pipiens pallens* [101], 50% in *T. castaneum* [102], and 60–68% mortality in *Lasioderma serricorne* [103]. In *H. armigera,* treatment with ds*AK* resulted in drastic reductions in body weight (17–38%), body length (11–27%), and pupation rate (43–49%) within five days of exposure [104]. Similarly, *P. xylostella* led to mortality rates of 25% with ds*AK*-plants, 23% with ds*β*-plants, and 30% with ds*AK-β*-plants, compared to 7.5% observed in wild-type plants [105].

In *N. lugens*, *AMPKα* knockdown reduced ATP levels and increased mortality [106]. In *D. melanogaster* and *B. mori*, 20-hydroxyecdysone (20E) activates the AMPK, antagonizing insulin/IGF signaling and slowing growth, demonstrating its role in hormonal regulation and energy balance [107]. In *T. castaneum*, suppression of *AMPK* disrupted lipid and carbohydrate metabolism (Figure 2) [108].

Silencing of the *AChE* gene experienced 100% mortality in *B. tabaci* [70], 22–43% in *Plutella xylostella*, *Tuta absoluta*, and *L. decemlineata* [109,110,111], and 60–68% in *Diaphorina citri* and *H. armigera* [112,113]. Furthermore, *Scirpophaga incertulas* exhibited reduced larval growth with a 15–20% increase in mortality [114], while *Pentalonia nigronervosa* showed 47–76% population reductions across Cavendish Williams, Gonja Manjaya, and Orishele banana cultivars [115] (Table S1).

In *P. xylostella,* dsRNA targeting *Pxace1* and *Pxace2* resulted in 34% and 23% larval mortality respectively [116]. Silencing of *ace* genes induced 25% mortality in *Chilo suppressalis* [117], 26% in *Bombyx mori* [118], 60% in *D. citri* [112] and 53% *H. armigera* [119]. In *T. castaneum ace,* knockdown disrupted metamorphosis, reproduction, and increased insecticide susceptibility [120].

#### 3.1.4. Detoxification- and Adaptation-Related Genes

Silencing *CYP* decreased larval tolerance to flavone in *H. armigera* [121] and increased mortality by 27–55% in *Spodoptera exigua* [122]. Similarly, targeting *CYP* increased nymph mortality in *B. tabaci* [123], reduced cuticular hydrocarbons (CHCs), enhanced cuticle permeability, and compromised insecticide tolerance in *B. germanica* [124]. In *L. migratoria*, *CYP* silencing enhanced susceptibility to desiccation and insecticide toxicity [125]. Notably, knockdown in *Agasicles hygrophila* inhibited larval molting, delayed development, and suppressed reproduction, highlighting the functional importance of *CYP* genes and potential in controlling pests [126].

RNAi targeting *GST* genes increased mortality in various pests: 50% in *Ostrinia furnacalis* with DIMBOA [127], 69% in *L. migratoria* with malathion [128], 23% in *D. citri* with thiamethoxam [129], and 47% in *Grapholita molesta* with imidacloprid [130]. Additionally, *GST* knockdown in *N. lugens* and *B. tabaci* enhanced pesticide susceptibility [131,132].

#### 3.1.5. Behavioral and Physiological Regulation Genes

*Neuropeptide F receptors* (*NPFR*) regulate feeding, metabolism, and reproductive behaviors in insects. RNAi targeting *NPFarFR/NPF* genes in various pests, including *O. furnacalis* [133], *G. molesta* [134], *L. migratoria* [135], *A. pisum* [136], and *Dendroctonus armandi*, reduced feeding, growth, and reproductive capacity, highlighting their potential as pest control targets [137].

In summary, rational RNAi target selection increasingly prioritizes those genes that are highly conserved, essential and readily accessible to RNAi machinery. Future research should combine bioinformatics screening, multi-gene targeting and stage-specific expression profiling to improve RNAi efficacy under field conditions.

However, the identification of conserved “core targets” involves an important trade-off. Highly conserved genes may increase the probability of strong RNAi phenotypes across pest taxa, but they may also increase the risk of unintended effects on closely related non-target organisms if sequence similarity is high [22]. Therefore, broad-spectrum RNAi target selection should be balanced with species-specific dsRNA design, bioinformatic screening against beneficial insects, and experimental validation in representative non-target species.

### 3.2. Quantitative Summary of the Key Targeted Genes

To complement our qualitative review, we conducted a descriptive summary of the RNAi efficacy data compiled in Table S1. For each study, we extracted the target gene and gene family, delivery method, and explicit mortality percentages reported in the original manuscripts. Only percentages associated with mortality, lethality, or survival reduction were included. Because of the heterogeneity in pest species, developmental stage, RNAi construct, and experimental design, we restricted analyses to descriptive rather than formal meta-analysis. These values should be interpreted as descriptive empirical baselines rather than formal estimates of treatment effect. Direct comparison among studies is limited by differences in insect order, species, developmental stage, dsRNA dose and length, exposure duration, target gene region, delivery method, and mortality endpoint. Because many studies did not report variance estimates, sample sizes, or standardized control-corrected mortality values, formal effect size estimation and confidence intervals were not consistently feasible. Therefore, medians, ranges, and interquartile ranges were used to summarize broad patterns while avoiding overinterpretation.

From 96 independent mortality observations across studies, the overall median reported mortality was 74% (IQR = 50–100%, range 18–100%).

#### 3.2.1. Delivery Method Comparison

**Figure 3:**
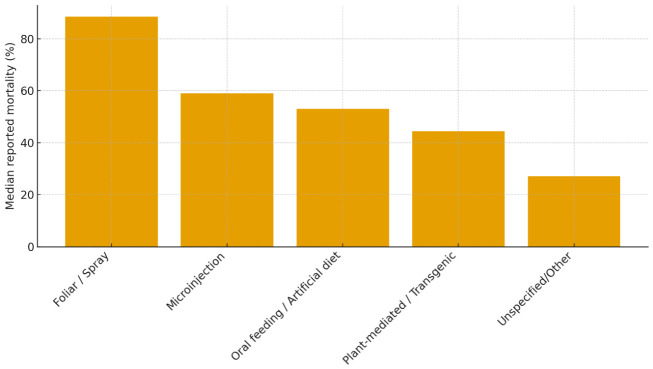
Median reported mortality by delivery method across RNAi studies (compiled from Table S1). Bar plot showing the median percentage mortality achieved via different delivery strategies. Values represent descriptive medians (not weighted meta-analysis) derived from 96 mortality observations.

Mortality medians varied among delivery routes (Figure 3; Table 1). Foliar/spray applications showed the highest median efficacy, 90–100%, particularly when dsRNA was adequately protected or formulated. Microinjection consistently produced high lethality with a median of approximately 80%, although it is not field-scalable. Oral/artificial diet feeding produced more variable outcomes with a median of 65%, while plant-mediated/transgenic approaches showed a median of approximately 60%.

#### 3.2.2. Gene Family Comparison

Several target classes emerged as promising high-lethality candidates across species (Table 2), including *ACT*, *V-ATPase*, *CHS/CHI*, *EcR*, *AChE*, and *COPI* components. These genes represent potential cross-species core targets for RNAi-based pest control. In contrast, *CYP*s and *GST*s mainly enhanced susceptibility to chemical insecticides, making them useful for integrated resistance management rather than as standalone lethal targets.

**Figure 4:**
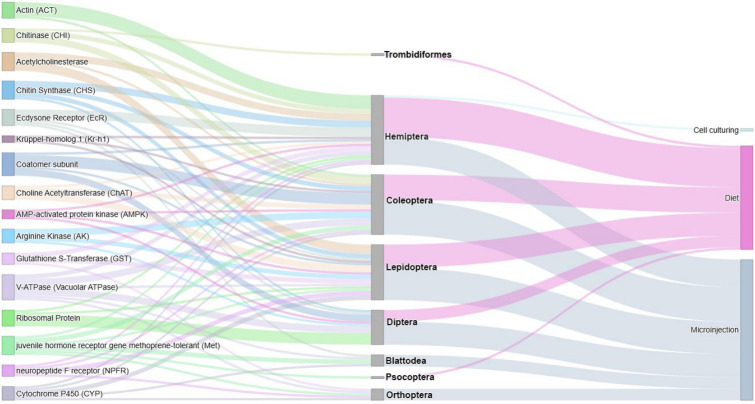
Alluvial mapping of RNAi target genes across insect orders and delivery methods (compiled from Table S1). Sankey diagram showing relationships between gene families (**left**), insect orders (**middle**), and delivery strategies (**right**). Link widths represent the number of mortality observations.

Overall, oral, plant-mediated, and foliar/spray delivery routes appear more relevant for field deployment, with foliar/spray formulations showing particular promise when supported by protective carriers (Figure 4).

We emphasize that these quantitative outcomes are descriptive: species identity, life stage, dsRNA design, and exposure conditions introduce heterogeneity that precludes formal meta-analytic effect size estimation. Nevertheless, compiling ranges and medians across >90 mortality observations provides a valuable empirical baseline. This approach highlights the targets most likely to generalize across taxa and the delivery strategies most suitable for field translation, thereby filling the gap between molecular RNAi studies and agronomic application.

## 4. Barriers and Roadmap for the Commercialization of RNAi-Based Technologies

**Figure 5:**
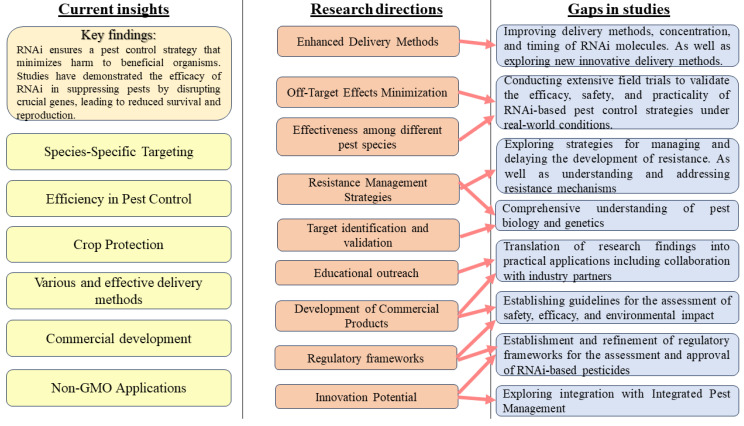
Current insights and future directions for RNAi in crop protection. Mechanisms and applications of RNAi in targeting agricultural insect pests, summarizing the latest key findings related, the research progress and its potential benefits. It also outlines key research directions and presents the main gaps in current studies.

The commercialization of RNAi-based biopesticides faces several challenges, including high production cost, instability of RNA molecules in the environment, variability in RNAi efficiency across species, regulatory hurdles, and limited field efficiency under varying environmental conditions (Figure 5).

### 4.1. Environmental and Molecular Barriers to RNAi Efficiency

Naked dsRNAs are highly vulnerable to degradation in agricultural environments due to nucleases, UV radiation, temperature fluctuations, and soil pH [17,18,19,20]. Although rapid degradation reduces environmental and biosafety concerns, it limits effective delivery to target pests [138,139]. Competition with endogenous RNA pathways and viral suppressors of RNAi may also contribute to inconsistent RNAi responses [140]. In this context, environmental degradation should be distinguished from molecular barriers operating inside the insect. Molecular barriers include competition with endogenous RNA pathways, viral suppressors of RNAi, inefficient intracellular processing, and variation in core RNAi machinery, all of which can reduce the intensity or consistency of gene silencing.

### 4.2. Cellular and Organismal Barriers

Inefficient cellular uptake of dsRNA is another major limitation. Some insect species of Diptera, Mecoptera and Siphonaptera show inefficient cellular uptake of dsRNA due to the absence or low expression of dsRNA transporter protein SID-1, which limits RNAi activation [141]. Endosomal entrapment of dsRNA and limited systemic spread of the RNAi signal further reduce its efficacy in many pest species [142].

### 4.3. Physiological Barriers

RNAi efficiency in insects is also influenced by several physiological factors. Rapid degradation of naked dsRNA by double-stranded ribonucleases (dsRNases) present in the gut, saliva, and hemolymph are a major factor that limits effective gene silencing [93]. Studies have shown that harsh gut conditions, particularly the presence of digestive enzymes and high-alkaline pH (9–10.5), accelerate the dsRNA degradation before it reaches the target cells. Additionally, the negatively charged peritrophic matrix composed of chitin and glycoproteins restricts dsRNA mobility and impedes its uptake into the gut epithelial cells of insects [143]. In addition, the developmental stage, tissue type, and immune responses may influence the level of gene silencing [144].

For example, studies in *B. mori* have shown that Toll-related receptors such as *BmToll9-1* and *BmToll9-2* can respond to exogenous dsRNA, supporting the idea that dsRNA may activate immune recognition pathways in insects [145,146,147]. Such immune activation could influence dsRNA stability, uptake, systemic spread, and the final efficiency of gene silencing.

The importance of dsRNases in limiting RNAi efficiency was first highlighted in insects through the isolation and characterization of a dsRNA-degrading nuclease from the digestive juice of *B. mori*, which showed that extracellular or gut-associated nucleases can degrade dsRNA before it reaches target cells [148].

### 4.4. Strategies to Overcome RNAi Barriers

To overcome these formidable challenges, several innovative approaches are being developed to enhance dsRNA delivery and stability in RNAi-based applications. One promising approach involves the development of nano-delivery methods to address dsRNA instability in spray-induced gene silencing (SIGS) [149,150,151,152]. These nanoparticle complexes encapsulate dsRNAs, effectively protecting them from nuclease and alkaline degradation, thereby enhancing their stability and RNAi efficacy. Therefore, owing to their diverse structures, distinct functional properties, low cytotoxicity, and strong adhesion capacity, these complexes have become indispensable vehicles for dsRNA delivery. However, to harness the full potential of these delivery systems, rigorous attention to their structural design and engineering is essential [153].

In recent years, multiple nanocarriers have been strategically engineered to overcome the challenges associated with genetic transformation, nucleic acid delivery, effective RNAi, dsRNA stability, controlled release and targeted delivery into pest cells for gene silencing [154,155]. The most representative classes of nanoparticles that have been designed for dsRNA delivery include natural organic carriers chitosan nanoparticles (CNPs), liposomal-based liposomes, inorganic nanomaterials star polycations (SPcs), layered double hydroxides (LDHs), carbon quantum dots (CQDs), synthetic polymers guanylated polymers (GNPs), peptide-based nanocarriers cell-penetrating peptides (CPPs) and branched amphiphilic peptide capsules (BAPCs) [156,157].

Recent studies with CNPs and their derivatives demonstrated effective gene silencing, growth inhibition, and mortality across pests including *A. aegypti* [158], *C. suppressalis* [159], *Spodoptera frugiperda* [160], *B. mori* [161], *H. armigera* [162], and *Frankliniella occidentalis* while minimizing off-target effects [163]. Similarly, liposome nanoparticles enhanced dsRNA delivery, and enabled significant gene knockdown and mortality in *D. suzukii* [57], *C. suppressalis* [159], *Earias vittella* [164], and *Rhipicephalus haemaphysaloides*, demonstrating their cross-taxa applicability, and suggesting their broad potential for insect and vector control. However, limitations such as poor stability under high gut pH, water insolubility, and low transfection efficiency are being addressed through chemical modifications and particle stabilization [165].

Similarly, inorganic carriers such as SPcs and CQDs showed promising stability, cellular uptake, and strong gene knockdown across lepidopterans, hemipterans, thrips and aphids [166,167]. LDH nanoparticles enable up to 90% gene silencing with marked physiological effects in sap feeding insects such as *Panonychus citri*, *Diaphorina citri*, and *A. gossypii* [168], and underground pests like *Holotrichia parallela* [169]. In addition, sprayable formulations based on LDH nanoparticles, such as BioClay^TM,^ have been developed at the University of Queensland [170]. This innovation facilitates dsRNA adhesion to plant surfaces and enhances nucleic acid uptake [21,171,172].

GNPs are a promising type of nanocarrier and establish the stable ionic interactions with the dsRNA that ensures the alkaline stability of the latter in the midguts of insects, leading to longer stability of the dsRNA and increased cell uptake [173]. Recent studies have shown that GNP-dsRNA complexes achieved 98% gene silencing and 53% larval mortality in *S. exigua*, and over 80% knockdown of target genes in *S. frugiperda* with low toxicity [173,174]. These findings highlight the strong potential of GNPs to overcome key physiological barriers that typically limit the oral RNAi efficacy in insects.

Peptide-based nanocarriers, including CPPs and BAPCs, provide strong electrostatic binding to dsRNA, facilitate cellular uptake and shield from degradation. Although CPPs improve transmembrane delivery and endosomal escape of dsRNAs, their effectiveness via oral RNAi is often limited by rapid intracellular degradation. In contrast, BAPCs with stable bilayered structures overcome this limitation, resulting in up to 97% gene silencing and substantial mortality in pests such as *A. pisum*, *T. castaneum* and *Popillia japonica* [175,176]. Collectively, these adaptable carriers offer versatile and effective platforms for RNAi, particularly when physiological barriers impede conventional dsRNA uptake across diverse insect species.

Emerging strategies also include integrating RNAi expression elements into novel nanocarriers via branched-strand PCR to produce stable short hairpin RNAs (shRNAs) within cells, enabling effective and sustained gene silencing [138,177]. In addition, artificial nanovesicles (AVs) have been employed for extending plant resistance to pathogens like *Botrytis cinerea* [178]. These advances in nanomaterials and nucleic acid nanotechnology represent critical developments for the successful design and implementation of RNAi-based biopesticides.

Along with non-transgenic delivery methods, a transgenic approach known as host-induced gene silencing (HIGS) is a highly efficient way to address numerous limitations of RNAi delivery, including the instability of dsRNA, gut degradation, low uptake, limited persistence in the field, and inconsistent delivery in pests. Crop plants are genetically modified to facilitate the production of the stable expression of the crop plant using dsRNA, hairpin RNA or artificial microRNAs against important pest genes. When fed, pests digest these RNAs, which allows the sustained systemic silencing of genes without sprays or complicated compounds. This gives long-term protection, which may cause high mortality, decrease in growth or decrease in reproduction. One of the commercial success stories is that of SmartStax PRO maize, which utilizes dsRNA targeted to the *DvSnf7* gene in *D. virgifera virgifera* with the assistance of *Bt* toxins to achieve superior efficacy and resistance control [179]. HIGS has also been applied to lepidopterans such as *H. armigera* in cotton [180] and hemipterans such as aphids [181,182]. Chloroplast expression can usually enhance the performance, as it yields greater amounts of intact long dsRNA, particularly with chewing insects [183]. Despite its ability to provide better reliability in some systems, HIGS has regulatory challenges, problems with GMO acceptance, increased costs in development, crop-specific restrictions, and the risks of resistance [184,185]. Nevertheless, it can be used alongside non-transgenic approaches and enhance the general RNAi-based pest control toolkit.

However, cost-effective dsRNA/siRNA synthesis remains a major limitation for the commercial adoption of RNAi-based biopesticides. To address this, cost-effective mass production methods are being developed. Several industrial companies are exploring microbial-based biosynthetic systems as a solution. For instance, Renaissance Bioscience (Vancouver, BC, Canada) (https://www.renaissancebioscience.com/) has explored the use of *Saccharomyces cerevisiae* as a production and delivery platform for dsRNAs. This approach aims to express dsRNAs/siRNAs targeting genes of specific pest species in yeast, with the robust cell wall of yeast providing additional stability to the RNA-based biopesticides. The RNA-containing yeast cells are often processed into formulations such as sprays or granules, which are then applied directly on crops or agricultural environments. After pests come into contact with or ingest the treated plants, the silencing of critical genes leads to pest mortality or reduced fitness. In addition to yeast-based systems, conventional microbial chassis such as *Escherichia coli* and *Bacillus subtilis* are widely employed for dsRNA bio-manufacturing [186,187,188,189,190,191]. These microbial platforms offer scalable and efficient production systems, providing avenues for cost-effective RNAi-based pest control solutions.

### 4.5. RNAi-Based Biopesticides vs. Chemical Pesticides

RNAi-based biopesticides and chemical pesticides offer distinct approaches to managing agricultural pests (Table 3). RNAi technology stands out for its high target specificity, silencing pest-specific genes without harming non-target organisms or beneficial species [15,27]. In contrast, the broad-spectrum activity of chemical pesticides often impacts multiple organisms even across different insect orders at different levels (including lethal and sublethal effects). Such pesticides might also act by disrupting ecosystems by contributing to insect biodiversity decline and by disrupting the ecosystem functions supported by insect biodiversity [192,193,194]. The rapid environmental degradation of dsRNA/siRNA further reduces the long-term ecological risks of RNAi-based biopesticides. Conversely, chemical pesticides can persist in the environment, leading to the contamination of water, soil and plant residue and unintended consequences to the ecosystems [194]. However, RNAi-based biopesticides may require more frequent applications to reach acceptable pest control due to an often lower in-field efficiency [25].

Recent commercialization progress shows that RNAi-based pest control is moving from proof-of-concept studies toward marketable products. Commercial and near-commercial examples include plant-incorporated RNAi traits such as dsRNA-expressing maize targeting western corn rootworm, sprayable/formulated dsRNA products, and pollinator protection RNAi products such as Norroa. These examples demonstrate that RNAi commercialization is feasible, but still limited to a small number of pest–crop or pest–host systems because of challenges related to delivery, stability, cost, regulatory approval, and field consistency [195].

Given that insect pests have increasingly developed resistance to chemical pesticides, RNAi offers a novel mode of action at the molecular level, potentially slowing resistance evolution [196,197,198]. Moreover, RNAi aligns with the increasing demand for environmentally responsible pest management solutions. Chemical pesticides remain cost-effective and widely available, benefiting from decades of research and streamlined regulatory frameworks [199]. Despite these advantages, their broad-spectrum effects and persistent environmental impact raise several safety concerns to human health and non-target biodiversity [200].

Nevertheless, RNAi-based biopesticides also face sustainability challenges. These include the instability of dsRNA under field conditions, high production and formulation costs, variable efficacy among insect taxa, and possible resistance evolution through changes in dsRNA uptake or processing pathways, and regulatory uncertainty. Therefore, the environmental advantages of RNAi should be evaluated case by case rather than assumed universally.

RNAi-based technologies should not be considered standalone replacements for all pest management tools, but rather as components of broader ecologically-based pest management frameworks. Their greatest value may lie in integration with biological control, host–plant resistance, habitat management, monitoring-based decision thresholds, and reduced-risk chemical tools. Such integration would help preserve ecosystem services, delay resistance evolution, and align RNAi deployment with sustainable pest management rather than single-tactic pest suppression [201].

### 4.6. Regulatory and Environmental Biosafety Considerations

In addition to technical and physiological barriers, regulatory uncertainty remains a major constraint for the commercialization of RNAi-based biopesticides. Risk assessment frameworks increasingly require evidence on environmental persistence, non-target effects, exposure routes, and food/feed safety [202,203]. Although sequence-dependent off-target effects are usually considered the primary biosafety concern, ecological risk assessment should also consider indirect effects [204]. For example, dsRNA exposure may alter insect-associated microbiota, which can influence host development, immunity, nutrition, and ecological fitness [205]. Therefore, future RNAi risk assessments should integrate bioinformatic off-target screening with experimental evaluation of non-target organisms, microbiome-associated interactions, and broader ecosystem functions.

## 5. Conclusions and Future Perspectives

Post-transcriptional gene silencing via RNAi has emerged as a complementary approach to reduce crop losses associated with insect damage by selectively targeting insect pests in a species-specific manner with low risks to non-target organisms. Recent studies have demonstrated that direct pest lethality may not always be required for controlling the insect pests. For instance, some works reported that silencing genes responsible for pheromone receptors could disrupt mating communication or host–plant location behaviors, effectively controlling pest populations without killing insects [206,207,208]. RNAi also holds significant potential not only for addressing insecticide resistance, but also for improving insecticide application efficiency (reduced concentration and application frequencies). By silencing specific genes associated with resistance mechanisms, the susceptibility of pests to chemical insecticides can be restored, reducing the quantities of insecticides required and enhancing their in-field efficiency. These dual benefits contribute to more sustainable pest management practices and expand the utility of RNAi in agricultural crop protection [209]. Emerging innovations are expected to greatly enhance the applicability of RNAi in pest management. Nanotechnology-based carriers can protect dsRNA from degradation by nucleases, improve cellular uptake, and enable more targeted delivery in insects. Likewise, microbial platforms such as engineered *Bacillus thuringiensis* or symbiotic gut bacteria represent sustainable approaches for continuous in situ dsRNA production and delivery under field conditions. Despite its potential, RNAi is currently implemented as a practical plant protection strategy in the field only against a few insect pest species such as *D. virgifera virgifera*, *P. xylostella*, and certain species of aphids [14,210]. The development of commercially viable dsRNA-based insecticides requires further investigation before their widespread implementation becomes feasible [211,212].

This review thus provides a unique contribution by offering a gene-focused synthesis, serving as a practical roadmap for selecting cross-species RNAi targets. Unlike the earlier literature, our emphasis on integrating target genes with delivery strategies situates this work as a bridge between functional genomics research and agronomic practice. In doing so, we highlight the gene-focused synthesis and integration of target genes with delivery strategies that may facilitate commercialization and field deployment.

The successful adoption of RNAi-based biopesticides will depend on addressing evolving regulatory and biosafety concerns. In the United States, the Environmental Protection Agency (EPA) has released draft guidance emphasizing ecological safety, off-target effects, and non-target organism assessments. Similarly, the European Food Safety Authority (EFSA) has outlined principles for environmental risk evaluation, highlighting the importance of standardized protocols and data transparency. These frameworks reflect growing international recognition of RNAi technologies while ensuring that ecological and food safety concerns are adequately addressed. To gain public trust and regulatory approval, future research should prioritize studies on environmental persistence, potential effects on beneficial species, and delivery system biosafety. Early alignment with these evolving policies will be essential to accelerate commercialization and ensure the responsible deployment of RNAi as a next-generation pest management tool.

**Figure 6:**
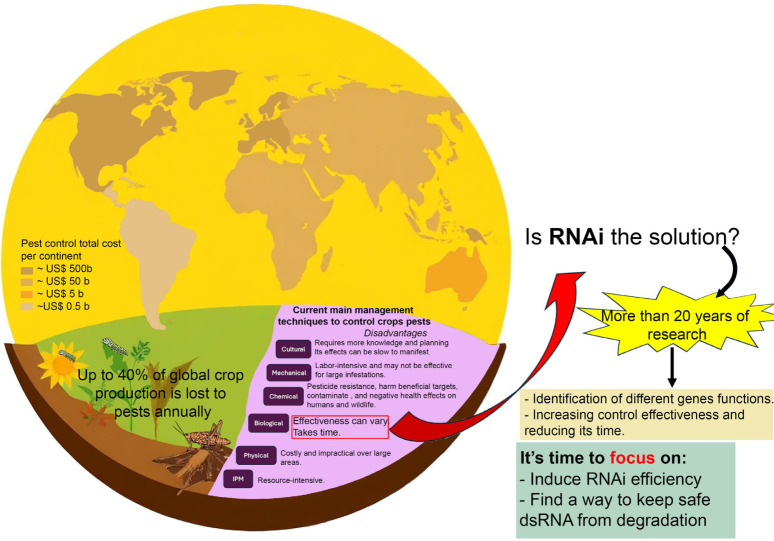
The figure highlights the global economic losses caused by insect pests, with a continental breakdown showing pest control costs. Limitations of the main current pest management techniques, including cultural, mechanical, chemical, biological, physical, and integrated pest management (IPM), are highlighted, including low efficiency, labor intensiveness, resistance development, and high resource requirements. The potential of RNAi technology for pest control is supported by over 20 years of research. Key advancements in RNAi include identifying gene functions, improving control effectiveness, and reducing time to achieve results. However, the current focus must shift toward addressing challenges associated with field application, efficient delivery methods, and enhancing the stability of dsRNA to fully realize RNAi’s potential in pest control.

Globally, crop losses caused by insect pests average approximately 20–30% of potential agricultural production, although in some cases, these losses can reach up to 40%, highlighting the significant impact of insect herbivory on global food security [213,214]. Future research must focus on unraveling the mechanisms underlying RNAi and assessing the potential risks associated with its application to ensure its safety and effectiveness (Figure 6). Advancing delivery methods is critical for enhancing the stability and uptake of RNAi molecules under field conditions, particularly in the face of environmental challenges. Additionally, developing integrated pest management strategies that minimize the evolution of resistance in targeted pests is essential for the long-term viability of RNAi-based approaches. A promising recommendation is the combined use of RNAi with microorganisms engineered to produce or deliver dsRNA. This could improve the stability and scalability of RNAi in field applications. For example, microbial formulations could serve as biocarriers, enhancing the persistence of dsRNA in the environment and facilitating its uptake by target pests. By addressing these challenges, RNAi-based pest control strategies can become a transformative tool for sustainable agriculture, offering targeted and eco-friendly solutions for protecting crops against insect pests. Future studies should address several concrete research questions. Firstly, which target genes combine high pest lethality with minimal sequence similarity to beneficial and non-target organisms? Secondly, which delivery systems provide the best balance between dsRNA protection, cost, scalability, and field persistence? Thirdly, how do environmental factors such as UV radiation, rainfall, temperature, pH, and plant surface chemistry affect dsRNA stability under realistic field conditions? Fourthly, how rapidly can insect pests evolve resistance through changes in dsRNA uptake, nuclease activity, or RNAi core machinery genes? Finally, how do RNAi applications affect non-target organisms, insect-associated microbiota, and broader ecosystem interactions? Addressing these questions will be essential for moving RNAi-based pest control from laboratory efficacy toward responsible and durable field deployment.

## Figures and Tables

**Table 1:** Summary of mortality outcomes by delivery method. Descriptive statistics (*n*, median, mean, minimum, maximum) for mortality observations compiled from Table S1. These values illustrate relative efficiencies of RNAi delivery strategies, highlighting the trade-off between laboratory efficacy and field scalability.

Delivery	Count	Median	Mean	Min	Max
Foliar/Spray	2	88.5	88.5	77	100
Microinjection	44	58.95	59	0	100
Oral feeding/Artificial diet	29	53	55.46	10	100
Plant-mediated/Transgenic	14	44.45	49.03	7.5	100
Unspecified/Other	7	27	32	18	55

**Table 2:** Summary of mortality outcomes by gene family (top 10 by median). Descriptive statistics (*n*, median, minimum, maximum) for mortality observations reported across gene families. Values are descriptive medians across heterogeneous studies.

Gene	Count	Median	Mean	Min	Max
*Ribosomal Protein* (*rp*)	4	84	77	40	100
*Actin* (*ACT*)	6	80	73	10	100
*Vacuolar ATPase* (*V-ATPase*)	10	80	74.1	28	100
*Chitin Synthase* (*CHS*)	8	70	67.525	44.2	100
*Ecdysone Receptor* (*EcR*)	9	68	67	23	100
*Acetylcholinesterase* (*AChE*)	9	60	57	17	100
*AMP-Activated Protein Kinase* (*AMPK*)	1	50	50	50	50
*Chitinase* (*CHI*)	8	49	48.525	38	59
*Coatomer Subunit*	12	48.335	56	0	100
*Glutathione S-Transferase* (*GST*)	5	47	45.54	28	68.9
*Arginine Kinase* (*AK*)	12	27.5	37.225	7.5	74.3
*Cytochrome P450* (*CYP*)	7	27	32	18	55
*Choline Acetyltransferase* (*ChAT*)	5	25	25.56	20	33.9

**Table 3:** Comparative analysis of RNAi-based pest control and chemical insecticides for crop protection. Key differences between RNAi-based biopesticides and conventional chemical insecticides used for insect pest management. Some challenges (e.g., variable efficacy and resistance development) are shared by both approaches, the underlying mechanisms and mitigation strategies differ substantially.

Aspect	RNAi-Based Pest Control	Chemical Insecticides
Target Specificity	Highly specific, targeting specific genes via sequence complementarity	Broad-spectrum or narrow-spectrum, affecting various organisms across orders
Environmental Impact	Generally lower impact on non-targets due to high specificity	May have non-specific ecological effects
Resistance Development	Potential for slower development; can be mitigated by multi-gene targeting	Often rapid development via target-site mutations or enhanced metabolism
Mode of Action	Sequence-specific post-transcriptional gene silencing via mRNA degradation	Direct interaction with proteins (e.g., enzyme inhibition, receptor modulation) disrupting biochemical pathways and behavior
Persistence	Transient (RNA degrades rapidly in environment)	Varies widely (some highly persistent, others short-lived)
Application Frequency	Often requires more frequent applications (topical methods)	Less frequent for many persistent formulations
Development Time	Research-intensive for target discovery and delivery optimization	Well-established, but new formulations needed due to resistance and market withdrawals
Costs	High R&D and production costs; improving with microbial biosynthesis	Established products with variable costs
Efficacy	Variable, depending on pest species, target gene selection, and delivery method; resistance can develop (though often slower)	Generally high initially but declines with resistance; also depends on pest, formulation, and application method
Regulatory Approval	Evolving framework	Established (but imperfect)
Non-target Organism Effects	Can be minimized via bioinformatics; risk mainly to closely related species	Often affects beneficial insects, vertebrates, and soil microbes
Compatibility with IPM	Highly compatible; integrates well with biological control	Can disrupt IPM by harming natural enemies
Public Acceptance	Concerns over genetic technologies, but perceived as more eco-friendly	Increasing concern over residues and pollution

## Data Availability

All studies reviewed have been cited. All databases accessed for information have been cited or their links and access dates are provided.

## Supplementary Materials

The following supporting information can be downloaded at: https://www.mdpi.com/article/10.3390/plants15121803/s1, Table S1: Summary of Genes Targeted by RNAi for Insect Pest Control. Includes Gene ID, Target Insect, Common Name, RNAi Effects and its corresponding Delivery Method.
