# Supplementary material for: RNAi Power Targets in Insect Pests: Beyond Functional Validation to Biopesticide Development Potential
Source: Plants (Basel). 2026 Jun 11;15(12):1803. doi: 10.3390/plants15121803 (PMC13306517; doi:10.3390/plants15121803)
Supplement: Supplementary file 1 [file plants-15-01803-s001.zip › plants-4341047-supplementary.pdf]

Table S1. Summary of Genes Targeted by RNAi for Insect Pest Control. Includes Gene ID, Target Insect, Common Name, RNAi Effects and its corresponding Delivery Method.

Gene Name	Target Insect	Common Name	RNAi Effects	Delivery Methods
Actin (*ACT*)	*Leptinotarsa decemlineata*	Colorado potato beetle	Third instar: 10 µg leaf-1/ 100% mortality 1.5 µf leaf-1/ 77% mortality // reduced weight	Foliar application
	Nezara viridula	Southern green stink bug	Nymphs: 100%	Direct injection
	Homalodisca vitripennis	Glassy winged sharpshooter	Altered phenotypic	Cell culturing
	Euscelidius variegatus	Variegated leafhopper	Adults: 80% mortality at day 14	Direct injection
	Bactericerca cockerelli	Cockerell's leafhopper	Adults: 80-90% mortality at day 6	Artificial diet / Direct injection
	Cimex lectularius	Bed bug	Oviposition reduction (to 10%) and elevated mortality in female adults (80% at day 21)	Injection
Vacuolar-type ATPase (*V-ATPase*)	*Henosepilachna vigintioctopunctata*	28-Spotted ladybug	50%, 85% and 100% larval lethality	Dietary introduction
	Locusta migratoria	Migratory locust	Severe molting defects in nymphs and a high mortality rate of > 90%.	Microinjection
	Liriomyza trifolii	Western flower thrips	Adults: 100% mortality after 120 H	Microinjection
	Pectinophora gossypiella	Pink bollworm	Mortality about 18-28%	Microinjection
	Sphenophorus levis	Granary weevil	Reduction in larval growth and survival (40-70% mortality)	Microinjection and diet (transgenic)
	Cimex lectularius	Bed bug	Reduced egg lying, reduced egg lying (to zero), and *dsvATPase-E* caused about 80% mortality	Microinjection
	Periplaneta fuliginosa	Smokybrown cockroach	Nymphal/nymphal molting defects with incomplete shedding of old cuticles, growth inhibition, as well as bent and wrinkled cuticles of thoraxes and abdominal segments	Microinjection
	Bemisia tabaci	Tobacco whitefly	Mortality 80%	Artificial diet
	Drosophila suzukii	Spotted wing Drosophila	Larvae and adults mortality 40-58%	Artificial diet / Direct injection
Ribosomal Protein (*rp*)	*Aedes aegypti*	Mosquito	>88% reduction in fecundity; mortality not evaluated	Microinjection
	Musca domestica	Common fly	Ovaries showed highly reduced provisioning and clutch reductions of 94–99%	Microinjection
	Drosophila melanogaster	Fruit fly	Embryonic or first-larval lethality, delay of larval development, defects in eye and wing morphology, and dramatically reduced the number of S2 cells	Microinjection
	Leguminivora glycinivorella	Soybean pod borer	Larvae: Mortality 90-100% at day 14, diminished body weight	Microinjection
	Bemisia tabaci	Tobacco whitefly	Mortality 80%	Artificial diet
	Drosophila suzukii	Spotted wing drosophila	Larvae and adults mortality 30-40%	Artificial diet / Direct injection
Chitin synthase (*chs*)	*Phthorimaea operculella*	Potato tuber moth	Larvae mortality about 50-70% and phenotype abnormalities	Microinjection
	Henosepilachna vigintioctopunctata	28-Spotted ladybug	Larvae: Increases mortality and decreases molting rate (80%)	Dietary introduction
	Acyrthosiphon pisum	Pea aphid	A 44.7% mortality rate and a 51.3% moulting rate were observed 72 h after injection into fourth-instar nymphs; 44.2% deformity rate among newborn nymphs was obtained upon ingestion	Microinjection/ ingestion
	Aphis gossypii	Cotton aphid	Mortality 60-70%	Dietary introduction (artificial diet)
	Anthonomus grandis	Cotton boll weevil	Mortality 100% on day ten. Total number of eggs reduced till zero	Microinjection
	Chilo suppressalis	Rice stem borer	Inhibited the molting and pupation	Microinjection
	Anopheles gambiae	African malaria mosquito	Larval mortality 80%	Artificial diet

Ecdysone receptor (*EcR*)	*Nilaparvata lugens*	Brown planthopper	Mortality 90% / offspring reduction of 44.18-66.27%	Diet (transgenic)
	Blattella germanica	German cockroach	Molting defects, lower circulating ecdysteroid levels and defects in cell proliferation in the follicular epithelium. Knockdown nymphs survive to the adult stage // High mortality, wing abnormalities	Microinjection
	Bemisia tabaci	Whitefly	Mortality 100% day ten	Diet (transgenic)
	Helicoverpa armigera	Cotton bollworm	Larval mortality 50-60%; Variation in pupal weight, pupal deformities and total number of eggs layed (about 50% less)	Artificial diet
	Sitobion avenae	Grain aphid	Mortality 70-80%	Artificial diet
	Apolygus lucorum	Mirid bug	Third-instar nymphs with siRNAs resulted in phenotypic defects including arrested development, low weight of fifth-instar nymph and high mortality (70% app) compared with the controls	Microinjection
	Zeugodacus cucurbitae	Melon fly	About 68% of larvae fed with a ds*Zc EcR* -treated diet failed to enter the pupal stage and died. In addition, *Zc EcR* knockdown dramatically reduced pupal weight (by 3.24 mg on average) and fecundity (by about 23%).	Artificial diet
Acetylcholinesterase (*AChE*)	*Bemisia tabaci*	Whitefly	Mortality 100% day four	Diet (transgenic)
	Plutella xylostella		2nd instar larvae: Mortality 22 and 39%; phenotype effects	Microinjection
	Scirpophaga incertulas	Yellow stem borer	The reduced larval length and weight, increased mortality (15-20% more than control)	Diet (transgenic)
	Pentalonia nigronervosa	Banana aphid	When aphids were maintained on elite transgenic events, there was a 67.8%, 46.7%, and 75.6% reduction in aphid populations growing on Cavendish Williams, Gonja Manjaya, and Orishele cultivars, respectively, compared to those raised on nontransgenic control plants.	RNAi through transgenic expression of *AChE* dsRNA in banana and planttain plants
	Tuta absoluta	South american tomato pinworm	2 and 5 µg of dsRNA were able to reduce the gene expression in a range of 62.7-75.4%, inducing a maximum mortality rate of 92.59%.	Microinjection
	Leptinotarsa decemlineata	Colorado potato beetle (CPB)	Interference of *Ldace1* in CPB adults caused a significant increase in mortality (43%) as early as three days post-injection (p.i.), suggesting the essential role of *Ldace1*	Microinjection
	Diaphorina citri	Asian citrus psyllid	Treatment with dsRNA-*AChE* increased the mortality percentages of both nymphs and adults of *D. citri*. The mortality percentage increased with the increase in the concentration of applied dsRNA-*AChE*, and the highest mortality (> 60%) was observed at the highest applied concentration (125 ng/mu l).	Microinjection
	Helicoverpa armigera	Cotton bollworm	The *S. tuberosum* plants transformed with TRV-VIGS expressing *AChE* exhibited higher mortality > 68% than the control plants 17 %, recorded ten days post-feeding and significant resistance in transgenic (transient) plants was observed.	Plant mediated
Cytochrome P450 (*CYP*)	*Helicoverpa armigera*	Cotton bollworm	Knockdown of *HaCncC* significantly inhibited expression of flavone-induced *CYP321*A1 and resulted in a decrease in flavone induction of *CYP321*A1. *HaCncC* knockdown significantly reduced the tolerance of *H. armigera* larvae to flavone.	Microinjection
	Spodoptera exigua	Beet armyworm	RNAi-mediated silencing of *CYP9A10* further increased mortality by 18%, 26% and 35% at 48 h and by 27%, 43% and 55% at 72 h when larvae were exposed to diets containing chemicals as compared to the control.	
	Bemisia tabaci	Whitefly	RNAi knockdown of *CYP306A1* increased the mortality of nymphs after treatment with IMD in bioassay, suggesting a pivotal role of *CYP306A1* in conferring IMD resistance in the nymph stage. Additionally, our metabolism experiments in vivo showed that the content of IMD reduced by 20% along with cytochrome P450 reductase and heterologously expressed *CYP306A1*, which provides additional evidence for the important function of *CYP306A1* in metabolizing IMD that leads to the resistance.	Bioassay
	Blattella germanica	German cockroach	RNAi-mediated knockdown of *CYP4G19* significantly decreased its expression and caused a reduction in CHCs. Meanwhile, *CYP4G19* suppression resulted in a non-uniform array of the lipid layer, enhanced cuticle permeability, and compromised insecticide tolerance.	Microinjection

	Locusta migratoria	Migratory locust	Suppression of *LmCYP303A1* expression by RNAi caused a lethal phenotype with molting defect from nymph to nymph. In addition, *LmCYP303A1* RNAi resulted in locusts being more susceptible to desiccation and to insecticide toxicity.	Microinjection
	Agasicles hygrophila	Alligator weed flea beetle	Knockdown of *AhCYP307A2* or *AhCYP314A1* significantly inhibited larval molting, impaired last instar larva- pupa-adult transition, delayed ovarian development, and stopped egg production (i.e., no eggs were laid).	Microinjection
Coatomer subunit (*COP*)	*Nezara viridula*	Southern green stinkbug	Significant mortality of 43% was observed after 14 days of treatment with *dsαCop*	Oral feeding
	Brassicogethes aeneus	Pollen beetle	Beetles injected with dsRNA targeting α*COP* (at 0.14 µg/mg) showed 88% and 100% mortality at 6 and 10 days post-injection, respectively; where by the same time after dietary exposure, 43%–89% mortality was observed in the 3 µg dsRNA/µL treatment, though the effect was concentration-dependent.	Oral feeding & Microinjection
	Tuta absoluta	Tomato leafminer	Oral exposure of *T. absoluta* to *dsTa-αCOP* resulted in 50% mortality.	Oral feeding
	Aedes aegypti	Yellow fever mosquitoes	Study found that decreased expression of the γCOPI coatomer protein led to 89% mortality in blood-fed mosquitoes by 72 h postfeeding compared with 0% mortality in control dsRNA-injected blood-fed mosquitoes	Microinjection
	Diabrotica virgifera virgifera	Western corn rootworm	*Sec23* as RNAi target for in planta rootworm control. High mortality in exposed adult and larvae and moderate sublethal effects in the offspring of females exposed to *Sec23* dsRNA LC25	Microinjection
	Drosophila melanogaster	Fruit fly	COPI depletion resulted in the failure of cytokinesis, through disrupted accumulation of essential proteins and lipid components at the cleavage furrow region. Furthermore, it caused a reduction in the number of overlapping central spindle microtubules, which are essential for cytokinesis.	Microinjection
	Colaphellus bowringi	Cabbage beetle	Knockdown of *Sar1*, *Sec23* and *Sec24* suppressed feeding and increased mortality to 26.67%, 46.67%, and 42.22%, respectively.	Microinjection
	Henosepilachna vigintioctopunctata	28-Spotted ladybug	In this study, *H. vigintioctopunctata* showed similar sensitivity to both types of *dsHvCOPIs* production. Ingestion of bacterially expressed dsHvαCOPI and *dsHvγCOPI* led to the highest mortality in both larvae and adults.	dietary RNAi
	Z. cucurbitae	Melon fly	*ZcCOPI-alpha* caused 59% of larval mortality	Oral feeding
Arginine Kinase (*AK*)	*Phyllotreta striolata*	Striped cabbage flea beetle	The feeding bioassays indicated that minute quantities of dsRNA greatly impaired the beetle's development. Ingestion of dsRNA not only significantly retarded the development and increased the mortality of adults, it also greatly reduced fecundity and fertility	Oral feeding
	Culex pipiens pallens	Northern house mosquito	The co-knockdown of *CpAK1* and *CpAK2* mediated by RNAi led to high mortality (74.3%) of adult female mosquitoes and decreased hatchability (59.9%).	Microinjection
	Lasioderma serricorne	Cigarette beetle	The mortality rates of *dsLsAK*-treated individuals increased to 60.0% and 67.5% at days 12 and 15, respectively. These mortality rates were significantly higher than those in control larvae. Among the dead larvae fed with *dsLsAK*, ~25% showed morphological abnormalities: ~15% individuals turned dark brown (phenotype 1) and ~10% turned entirely black	Oral feeding
	Helicoverpa armigera	Cotton bollworm	The two dsRNAs (*dsAK1* and *dsAK2*) also caused drastic reductions in body weight (38.43% and 17.37%, respectively), body length (26.73% and 11.23%, respectively) and pupation rate (48.89% and 42.95%, respectively) compared to the control on day 5.	Artificial diet
	Plutella xylostella	Diamondback moth	*P. xylostella* mortality rates were 25.0% when exposed to *dsAK* plants, 22.5% with dsβ plants, and 30.0% with *dsAK*-β plants, which were all higher than 7.5% for the wild-type plant.	Plant mediated RNAi
	Tribolium castaneum	Red flour beetle	The knockdown of *TcAK1* and *TcAK2* significantly decreased the lifespan of beetles treated with deltamethrin relative to the *dsEGFP* group (median survival time −27.3 h and −24.9 h, respectively), About 50% mortality	Microinjection
Choline Acetyltransferase (*ChAT*)	*Plutella xylostella*	Diamondback moth	Silencing of the *Pxace1* gene resulted in 33.9% mortality in *P. xylostella* larvae, while silencing of the *Pxace2* gene resulted in 22.9% mortality. Larvae that survived the gene knockdowns	Microinjection

			exhibited growth inhibition, including reduced weight and length, as well as physical malformations and impaired motor abilities	
	Chilo suppressalis	Rice stem borer	Silencing of *Csace1* or *Csace2* resulted in approximately 25% mortality rate, indicating both *AChE-1* and *AChE-2* are important for maintaining life in this insect.<br>- Knockdown of *Csace1* had major effects on larval growth inhibition, reduced larval weight and length, malformation, and motor disability, whereas silencing of *Csace2* had only minor effects, suggesting *AChE-1* has a more significant role in regulating larval growth and motor ability compared to *AChE-2*	Microinjection
	Tribolium castaneum	Red Flour Beetle	Knockdown of *ChAT* and Vesicular acetylcholine transporter genes in *T. castaneum* affects metamorphosis, reproduction, and insecticide susceptibility	Microinjection
	Bombyx mori	silkworm	Silencing the *BmAce2* gene resulted in higher mortality compared to silencing the *BmAce1* gene. *BmAce2* resulted in about 26% mortality, faster and higher than the 20% in the *siBmAce1*-treated group.<br>Silencing the *BmAce1* gene had a greater impact on motor control and development compared to silencing the *BmAce2* gene	Microinjection
	Diaphorina citri	Asian citrus psyllid	Silencing the *AChE* and *cholinesterase 2-like* (*ChE-2-like*) genes in *D. citri* nymphs increased the susceptibility of the emerged adults to the *AChE*-inhibiting insecticides chlorpyrifos and carbaryl, but did not increase susceptibility to the nicotinic *acetylcholine receptor* agonist imidacloprid. - Treating adult *D. citri* directly with dsRNA-*AChE* increased their susceptibility to chlorpyrifos and carbaryl, but did not affect their susceptibility to imidacloprid.	Oral feeding
	Helicoverpa armigera	Cotton bollworm	Silencing the *AChE* gene in *H. armigera* larvae using siRNA led to increased mortality, growth inhibition, reduced pupal weight, larval malformation, and drastically reduced fecundity. - The study suggests novel roles for the *AChE* gene in insect larval growth and development, and demonstrates that siRNA can be effectively delivered to insect larvae through their diet.	Oral feeding
Chitinase (*Cht*)	*Chilo partellus*	Maize stem borer	Targeting the *chitinase* gene in the maize stem borer (*Chilo partellus*) using RNAi technology resulted in developmental abnormalities and mortality in the larvae. Larvae exhibited various phenotypic distortion levels across developmental stages, and 53% mortality occurred in transgenic fed larvae compared to those fed on nontransgenic leaves	leaf insect feeding bioassays
	Acyrthosiphon pisum	Pea aphid	Silencing the *chitin synthase* (*CHS*) gene through RNAi led to a 44.7% mortality rate and a 51.3% disruption of molting in pea aphid nymphs. *CHS* gene silencing also impaired nymphal development, resulting in a longer development period and a 44.2% deformity rate among newborn nymphs.	plant-mediated feeding
	Aphis gossypii	Cotton-melon aphid	Knockdown of the *chitin synthase 1* (*CHS1*) gene via oral delivery of dsRNA-*CHS1* caused up to 59% mortality in third-instar nymphs of the cotton-melon aphid, *A. gossypii*. - Oral-delivery-mediated RNAi of *CHS1* significantly reduced the adult longevity (by approximately 38%) and fecundity (by approximately 48%) of the cotton-melon aphid.	Oral feeding
	Leptinotarsa decemlineata	Colorado potato beetle	RNAi knockdown of chitin synthase genes in *L. decemlineata* inhibits chitin biosynthesis and causes mortality, decreased longevity, and reduced fecundity. RNAi targeting *LdChSB* is more effective against young larvae of the Colorado potato beetle, reducing their feeding and protecting potato plants	Oral feeding
	Panonychus citri	Citrus red mite	Silencing the *chitin synthase 1* (*PcCHS*1) gene in the citrus red mite, *P.citri*, using RNAi led to significant reductions in egg-laying potential and egg hatching rates. - Silencing the *PcCHS1* gene resulted in mortality of over 50% in larvae, protonymphs, and deutonymphs of *P. citri*. - Silencing the *PcCHS1* gene led to a reduction in chitin content in *P. citri* eggs and adults	Oral feeding
	Henosepilachna vigintioctopunctata	28-Spotted Ladybug	Knockdown of the *chitin synthase 1* gene (*HvCHS1*) in the pest insect *H. vigintioctopunctata* caused significant developmental defects, including arrested larval development, impaired larva-pupa-adult transition, and the production of stunted, deformed, or misshapen individuals. - Knockdown of *HvCHS1* also led to	Oral feeding

			defects in the insect's gut integrity, cuticle thickness, molting process, and tracheal system development.	
Glutathione S-Transferase (*GST*)	*Ostrinia furnacalis*	Asian corn borer	RNAi targeting the *GST* gene in Asian corn borer increases mortality, demonstrating its potential for pest control. Feeding asian corn borer larvae with dsRNA targeting *OfGST1* and the plant toxin DIMBOA resulted in over 50% mortality.	Oral feeding
	Locusta migratoria	Migratory locust	Suppressing the expression of the GST enzyme *LmGSTE4* in locusts increases their mortality when exposed to the insecticide malathion. Malathion bioassays of *L. migratoria* after the expression of *LmGSTE4* was suppressed by RNAi showed increased insect mortality from 33.8% to 68.9%.	Microinjection
	Grapholita molesta	Oriental fruit moth	Knockdown of two *GST* genes in *G. molesta* increased mortality from imidacloprid treatment, indicating GSTs play a key role in imidacloprid metabolism. Knockdown of *GmGSTD2* and *GmGSTD3* by RNAi, increased the mortality of *G. molesta* from 28% to 47% following imidacloprid treatment.	Oral feeding
	Nilaparvata lugens	Brown planthopper	Knockdown of seven *GST* family genes of brown planthopper increases its sensitivity to gramine. The GST activity is regulated by the expression of three key genes (*NlGST1-1*, *NlGSTD2*, and *NlGSTE1*), as silencing of these genes significantly inhibits this enzyme's activity. The current study identifies a few key *GST* genes involved in the detoxification of gramine in brown planthopper.	Oral feeding
	Bemisia tabaci	Silverleaf whitefly	Knockdown of *GST-d7* in *B. tabaci* MED but not in *B. tabaci* MEAM1 resulted in a substantial increase in the mortality of imidacloprid-treated adults.	Oral feeding
Neuropeptide F receptor (*NPFR*)	*Ostrinia furnacalis*	Asian corn borer	Inhibits larval feeding, growth and development,	Star polycation nanocarrier-based double-stranded RNA (dsRNA) delivery system.
	Grapholita molesta	Oriental fruit moth	After *GmolNPF1* knockdown by RNAi, the feeding amount, weight, body size and drill holes rate of larvae decreased significantly, and the number of larvae that ate food within 5 min declined significantly.	nanocarrier-mediated transdermal dsRNA delivery system
	Locusta migratoria	Migratory locust	Downregulation of *LmiNPF1* inhibited locust feeding behavior	Microinjected
	Acyrthosiphon pisum	Pea aphid,	Significantly reduced their food intake, reduce reproduction	Microinjected
	Dendroctonus armandi	Chinese white pine beetle	Reduced their food intake, caused increases of mortality and decreases of body weight, and also resulted in a decrease of glycogen and free fatty acid and an increase of trehalose.	Microinjected
Juvenile hormone receptor gene *methoprene-tolerant* (*Met*)	*Sogatella furcifera*	White-backed planthopper	Silencing of *SfMe*t substantially reduced the expression of *SfVg*, decreased yolk protein deposition and blocked oocyte maturation and ovarian development.	Microinjection
	Harmonia axyridis Pallas	Harlequin ladybird	Inhibited yolk protein deposition, and reduced fecundity using RNAi. the preoviposition period was significantly prolonged	Microinjected
	Liposcelis entomophila	Booklouse	Knockdown of both *LeMe*t and *LeKr-h1* significantly increased *L. entomophila* mortality.	Artificial diet.
	Coccinella septempunctata L.	Seven-spotted ladybug	Ovary development and vitellogenesis in *C. septempunctata* injected with *CsMet*-dsRNA were significantly delayed and fewer mature eggs were produced.	dsRNA injection
	Schistocerca gregaria	Female desert locusts	*SgMet* knockdown in females resulted in delayed display of copulation behavior with virgin males, an incapacity of adult *dsSgMet* injected female locusts to oviposit.	dsRNA injection
	Diploptera punctata	Cockroach	Silencing *Met* results in an arrest of basal oocyte development; vit ellogenin is no longer transcribed in the fat body and no longer tak en up by the ovary.	dsRNA injection
	Blattella germanica	German cockroach	Depletion of *Met* in this stage provoked deficiencies in wing growth and ecdysis problems in the imaginal moult.	dsRNA injection
	Helicoverpa armigera	Cotton bollworm	Knockdown of *HaMet* in final-instar larvae shortened the time of pupation, induced abnormal pupation, and dampened pupation rate. In female adults, *HaMet* depletion severely suppressed the transcription of *Vitellogenin* (*Vg*) and *Vitellogenin Receptor*	dsRNA injection

			(*VgR*), disrupted the Vg accumulation in fat body and the yolk protein uptake in oocytes, and finally led to an impaired fecundity.	
	Sogatella furcifera	White-backed planthopper	RNAi knockdown of *SfKr-h1* substantially reduced the transcription of *SfVg*, and arrested ovarian development.	dsRNA injection
Krüppel-homolog 1 (*Kr-h1*)	*Harmonia axyridis*	Harlequin ladybird	Knockdown of *HmMet* and *HmKr-h1* substantially reduced the transcription of *HmVg1* and *HmVg2*, inhibited yolk protein deposition, and reduced fecundity using RNAi.	Direct application on freezed samples
	Helicoverpa armigera	Cotton bollworm	Knockdown of *HaKr-h1* in fifth-instar larvae resulted in precocious metamorphosis from larvae to pupae. In female adults, depletion of *HaKr-h1* severely repressed the transcription of *Vg*, disrupted oocyte maturation and reduced the number of eggs laid,	Microinjection
AMP-activated protein kinase (*AMPK*)	*Nilaparvata lugens*	Brown planthopper	Knockdown of the AMPK catalytic subunit alpha in the rice pest *N. lugens* decreased the ATP level and increased mortality. - AMPK responded to the energy stress caused by the insecticide pymetrozine, activating energy metabolism pathways, but the ATP level still decreased, indicating ATP consumption exceeded production. - Knockdown of *NlAMPKα* in pymetrozine-treated *N. lugens* further decreased ATP levels and significantly increased mortality, allowing a 50% reduction in pymetrozine concentration while maintaining comparable efficacy.	Microinjection
	Drosophila melanogaster	Fruit fly	The hormone 20-hydroxyecdysone activates the AMPK-PP2A axis in the fat body, antagonizing insulin/IGF signaling and restricting growth rate	Microinjection
	Tribolium castaneum	Red flour beetle	*AMPK* suppression increased triglyceride levels and altered glucose-trehalose ratios, affecting lipid and carbohydrate metabolism	Microinjection
	Bombyx mori	Silkworm moth	The hormone 20-hydroxyecdysone activates the AMPK-PP2A axis in the fat body, antagonizing insulin/IGF signaling and restricting growth rate	Microinjection
